# Green Synthesis of Crystalline Silica from Sugarcane Bagasse Ash: Physico-Chemical Properties

**DOI:** 10.3390/nano12132184

**Published:** 2022-06-25

**Authors:** Ntalane S. Seroka, Raymond Taziwa, Lindiwe Khotseng

**Affiliations:** 1 Department of Chemistry, University of the Western Cape, Robert Sobukwe Rd, Private Bag X17, Bellville 7535, South Africa; 2 Department of Applied Science, Faculty of Science Engineering and Technology, Walter Sisulu University, Old King William Town Road, Potsdam Site, East London 5200, South Africa; rtaziwa@wsu.ac.za

**Keywords:** green synthesis, silica, tetrapropylammonium hydroxide, citric acid, L-cysteine hydrochloride monohydrate, sugarcane bagasse ash

## Abstract

Sugarcane bagasse South Africa is an agricultural waste that poses many environmental and human health problems. Sugarcane bagasse dumps attract many insects that harm the health of the population and cause many diseases. Sugarcane ash is a naturally renewable source of silica. This study presents for the first time the extraction of nanosilica from sugar cane bagasse ash using L-cysteine hydrochloride monohydrate acid and Tetrapropylammonium Hydroxide. The structural, morphological, and chemical properties of the extracted silica nanoparticles was cross examined using XRD, FTIR, SEM, and TGA. SEM analysis presents agglomerates of irregular sizes. It is possible to observe the structure of nanosilica formed by the presence of agglomerates of irregular shapes, as well as the presence of some spherical particles distributed in the structure. XRD analysis has revealed 2θ angles at 20, 26, 36, 39, 50, and 59 which shows that each peak on the xrd pattern is indicative of certain crystalline cubic phases of nanosilica, similar to results reported in the literature by Jagadesh et al. in 2015. The crystallite size estimated by the Scherrer equation based on the aforementioned peaks for ca-silica and L-cys-silica for the extracted particles had an average diameter of 26 nm and 29 nm, respectively. Furthermore, it showed a specific surface area of 21.6511 m^2^/g and 116.005 m^2^/g for ca-silica and L-cys silica, respectively. The Infrared (IR) spectra showed peaks at 461.231 cm^−1^, 787.381 cm^−1^ and 1045.99 cm^−1^ which corresponds to the Si~O~Si bending vibration, the Si~O~Si stretch vibration, and the Si~O~Si stretching vibration, respectively. This confirms the successful extraction of nanosilica from sugar cane bagasse ash. TGA analysis has revealed that the as received sugarcane bagasse has high loss on ignition (LOI) of 18%, corresponding to the presence of the unburnt or partial burnt particles, similar to results reported by Yadav et al. This study has shown that sugar cane bagasse ash is a natural resource of silica which should be harnessed for industrial purposes in south Africa.

## 1. Introduction

In nanotechnology, “green” synthesis has received a great deal of attention as a reliable, sustainable, and environmentally friendly protocol for synthesis for a wide range of nanomaterials for the development of solar cells. In essence, green synthesis is regarded as an important route to reduce the pernicious effects associated with the traditional methods of synthesis of nanoparticles for preparation of solar cell electrodes. It is well known that the four most important parameters for the synthesis of nanoparticles using the green protocol are the selection of an environmentally friendly solvent, a source of nanomaterials, a reducing agent, and a harmless material for stabilization [1,2,3]. 

Plants are known as natural chemical factories that are economical and have no conservation. Rodríguez-Félix, F. et al., reported on the biosynthesis of silver (Ag) using aqueous extract from waste such as safflower (*Carthmus tinctorius* L.), a well-known crop from the Northwest of Mexico. Parallel to environmental pollution reduction, industrial agro-waste and food research was further done on efficiency and environmentally friendly properties of phytochemicals from safflower by-product via the UPLC-DAD-MS method. In addition, researchers reported on the development and quality of food packaging which stems from the composites of zein films with no-ultrafiltred and ultrafiltred betalains extract(s) from the beetroot (*Beta vulgrains*) bagasse that is feasible for food packaging. Bio-inspired methods are emerging as viable options for producing highly valued silver nanoparticles while promoting sustainability. The green synthesis of Ag from agri-food waste extracts and by-products are flexible to manage, cost-effective, and possess potential application in health for antimicrobial and anticancer activity [4,5,6,7]. 

The synthesis of nanomaterials from agricultural waste is considered green because agricultural waste is an environmental hazard if not handled properly. For instance, sugarcane bagasse dumps attract many insects that harm the health of the population and cause many diseases. At the same time, sugarcane ash is a renewable source of silicon. Elemental analysis of sugar cane bagasse ash Fortunate et-al has revealed 71.49 wt% silica [8,9,10]. Another study reported on the sustainable and green synthesis of silver (Ag) nanoparticles with potential applications in medicine and the food industry as antibacterial agents. 

Thus, it is quite evident that conventional methods utilize very harsh and strong chemicals such as HCl, HNO_3_, H_2_SO_4_, NaOH and KOH. Mohd NK, et al., in 2017 reported the green synthesis of nanosilica from sugarcane bagasse using HCl and NaOH [1,11]. However, the fate of this hazardous chemical is of utmost importance, as it requires adequate care in disposal. Thus, several researchers have extensively explored other synthetic methods to get the raw materials of SiO_2_, as they are affordable and suitable in various applications [12]. A few decades ago, studies demonstrated that silica can be feasibly extracted from various agricultural wastes, such as rice straw, rice husk, corn cub, sugarcane bagasse and palm ash. Interestingly, there is a dearth of scientific articles that report on the synthesis of silica from sugarcane bagasse [13,14,15,16]. 

In this research, sugarcane bagasse is chosen as a natural source of silica nanoparticles. The advantages of this method are that it is less toxic and requires minimal chemical consumption. Furthermore, in this method metallic impurities will be removed by organic acid (pretreatment process). The absence of metallic impurities will lead to the high purity of silica nanoparticles production at the end of this research study. Taking advantage of agricultural waste utilization as a source of silica, this project is not only economically feasible, but also may provide a significant impact to environmental control [17,18].

The confirmation of desired functional groups vibration is determined by Fourier Transform Infrared (FTIR) spectroscopy, while size determination and morphology has been conducted using a scanning electron microscope (SEM). The structural characteristics were confirmed by X-ray powder diffraction (XRD). The thermal behavior was studied using a Thermogravimetric (TGA) analyzer, and the surface area of silica nanoparticles was determined using a BET surface area analyzer. Therefore, this work can be considered novel, because there are only a few published analyses related to sugarcane bagasse ash being used as a source of silica using organic bases.

## 2. Materials and Experimental Methods

### 2.1. Materials Used

The chemicals utilized in the preparation were citric acid ≥99.5%, L-cysteine hydrochloride monohydrate ≥98% and tetrapropylammonium hydroxide 1.0 M in H_2_O purchased from Sigma Aldrich. Sugarcane was procured from the Sugar Illovo South Africa Company. The synthesis was done using deionized water from the Milli-Q water purification system (Millipore, Bedford, MA, USA).

### 2.2. Preparation of Sugarcane Bagasse Ash (SCBA)

In a typical preparation procedure, sugarcane bagasse was soaked for a period of 24 h in double deionized water to remove any dust particles. The soaked sugarcane was then oven dried at 40 °C for a period of 6 h. The soaked, dried sugarcane was then burnt in open air to obtain a black ash sugarcane bagasse ash (SCBA). 

#### 2.2.1. Leaching Using Citric Acid

**Scheme 1:**
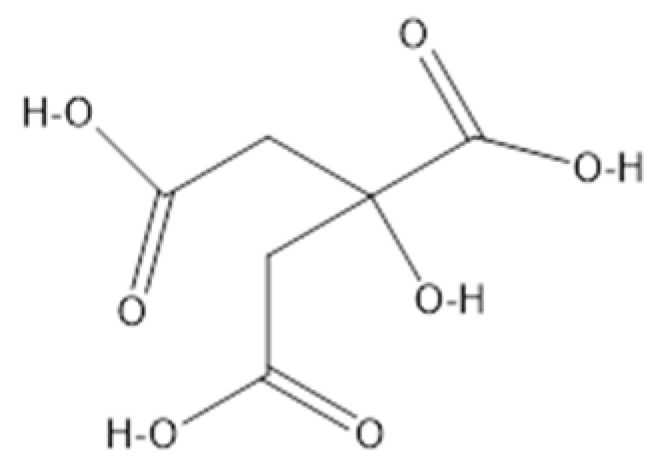
The structure of citric acid.

10.0 g of sugarcane bagasse ash was added to a 500 mL beaker of 5% citric acid solution, Scheme 1 showing the skeletal structure of citric acid. The black colored solution of sugarcane bagasse ash and 5% citric acid solution was then transferred into a 500 mL volumetric flask. The resulting solution in the 500 mL volumetric flask was first stirred at 450 rpm, which was subsequently refluxed at 70 °C for 24 h. The resulting pale yellow-green solution was then filtered using a Buchner funnel and 70 mm filter paper. The trapped precipitate was then washed with double deionized water, and pH of the decant was 3–5 until the pH of the supernatant reached 6.5. The resulting black sugar cane bagasse supernatant was then dried in an oven at 40 °C overnight and ground to a fine powder and stored in sample vials. It was subsequently labeled as SCBA-ca leached.

#### 2.2.2. Leaching Using Citric Acid L-cysteine Hydrochloride Monohydrate

**Scheme 2:**
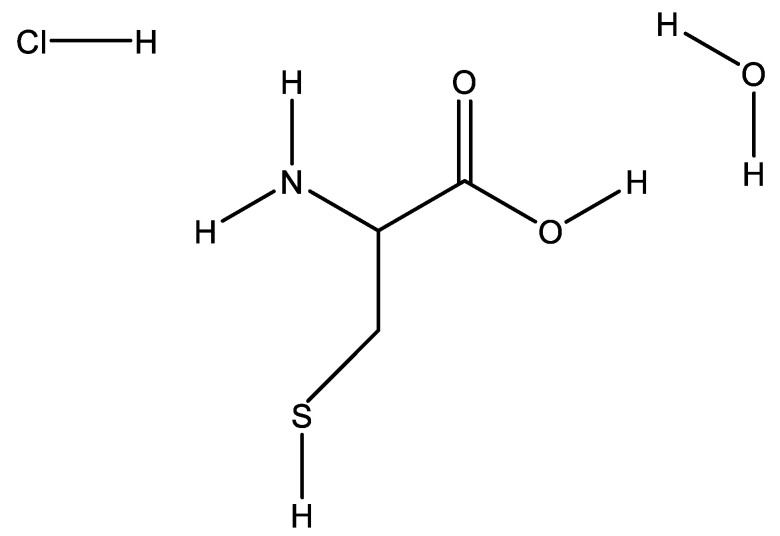
Show the structure of L-cysteine hydrochloride monohydrate.

The same process was repeated using 5% L-cysteine hydrochloride monohydrate (L cys):

Ten grams of sugarcane bagasse ash was added to a 500 mL beaker of 5% L-cysteine hydrochloride solution, Scheme 2 showing the skeletal structure of the acid. The black colored solution of sugarcane bagasse ash and % 5 L-cysteine hydrochloride solution was then transferred into a 500 mL volumetric flash. The resulting solution in the 500 mL volumetric flask was first stirred at 450 rpm, which was subsequently refluxed at 70 °C for 24 h. The resulting colorless-foam solution was then filtered using a Buchner funnel and 70 mm filter paper. The trapped precipitate was then washed with double deionized water, and the pH of the decant was 3–5 until the pH of the supernatant reached 6.5. The resulting black sugar cane bagasse supernatant was then dried in an oven at 40 °C overnight and ground to a fine powder and stored in sample vials. It was subsequently labeled as SCBA-L cys leached.

### 2.3. Extraction of Silica

#### 2.3.1. SCBA-CA Leached

In a typical procedure, the 2.0032 g SCBA-CA was placed in a ceramic crucible which was heated at 700 °C for 4 h (residence time) in a muffle furnace at 10 °C/min heating rate (gradient time) to burn away carbonaceous material and form crystalline silica. This resulted in the formation of an orange-brown product(s). Two mL of tetrapropylammonium hydroxide was then added to the orange-brown product shown in Equation (1) while stirring at 350 rpm for 1 h. The solution was then heated at 200 °C in air for the first 2 h and then further heated at 600 °C for 1 to burn away the excess carbon, nitrogen, and hydrogen, as shown in Equation (2). The heating resulted in the oxidation of C, H and N to form respective oxide(s). As result, silica oxide(s) was formed, and nano powders were labelled as CA-TPAH-silica.

(1) $${\left(SiO_{2}\right)}_{48}+\left[N{\left(C_{3}H_{7}\right)}_{4}\right]OH\to [N{\left(C_{3}H_{7}\right)}_{4}]OH{\left(SiO_{2}\right)}_{48}$$

(2) $$[N{\left(C_{3}H_{7}\right)}_{4}]OH{\left(SiO_{2}\right)}_{48}+\Delta \to {\left(SiO_{2}\right)}_{x}\left(s\right)↓+{\left(CO\right)}_{x}\left(g\right)↑+{\left(H\right)}_{x}\left(g\right)↑\;+{\left(NO\right)}_{x}\left(g\right)$$

#### 2.3.2. SCBA-L cys Leached

About 2.0032 g of SCBA-L cys was placed in a ceramic crucible and heated at 700 °C for 4 h (residence time) in a muffle furnace at 10 °C/min heating rate (gradient time) to burn away carbonaceous material and form crystalline silica. This resulted in the formation of a light orange-brown product(s). Next, 2 mL of tetrapropylammonium hydroxide (TPAH) was added to the orange-brown product whilst stirring at 350 rpm for 1 h, as shown in Equation (1). The solution was then heated at 200 °C in air for the first 2 h and then further heated at 600 °C for one hour to burn away the excess carbon, nitrogen, and hydrogen as shown in Equation (2). Heating resulted in the oxidation of C, H and N to form the respective oxide(s). As a result, silica oxide(s) was formed as nano powders labelled L cys-TPAH-Silica.

(3) $${\left(SiO_{2}\right)}_{48}+\left[N{\left(C_{3}H_{7}\right)}_{4}\right]OH\to [N{\left(C_{3}H_{7}\right)}_{4}]OH{\left(SiO_{2}\right)}_{48}$$

(4) $$[N{\left(C_{3}H_{7}\right)}_{4}]OH{\left(SiO_{2}\right)}_{48}+\Delta \to {\left(SiO_{2}\right)}_{x}\left(s\right)↓+{\left(CO\right)}_{x}\left(g\right)↑+{\left(H\right)}_{x}\left(g\right)↑\;+{\left(NO\right)}_{x}\left(g\right)↑$$

### 2.4. Structural, Morphological, and Thermal Characterization

The phase identification was obtained using X-ray diffraction (XRD) on a Bruker AXSD8 Advance instrument, (Ithemba Labs, South Africa) with Cu-Kα1 radiation, λ = 1.54050 A. The Bragg angle array was 2θ = 10–90 °C with a scanning step of 0.035°. The surface chemistry was evaluated using FTIR by identifying the bonding of elements inside the samples. The technique was undertaken at room temperature with a wavelength range 400–4000 cm^−1^ and phase composition was done using a PerkinElmer FTIR spectrometer, spectrum two. N_2_ physisorption at 77 K for textural properties (specific surface area (BET_SSA_), pore volume (Vp) and pore diameter (Dp). A thermographic analysis (TGA) using a PerkinElmer Simultaneous Thermal Analyzer STAA 8000 in order to measure the rate of mass loss over the temperature of sugarcane bagasse ash was done. The TGA was carried out in the presence of nitrogen gas, and a sample weight of 10 mg was heated from 30 to 800 °C with a constant heating rate of 10 °C/min. 

## 3. Results and Discussion

### 3.1. Characterization of the Synthesized Materials

#### 3.1.1. Powder X-ray Diffraction Analysis

**Figure 1:**
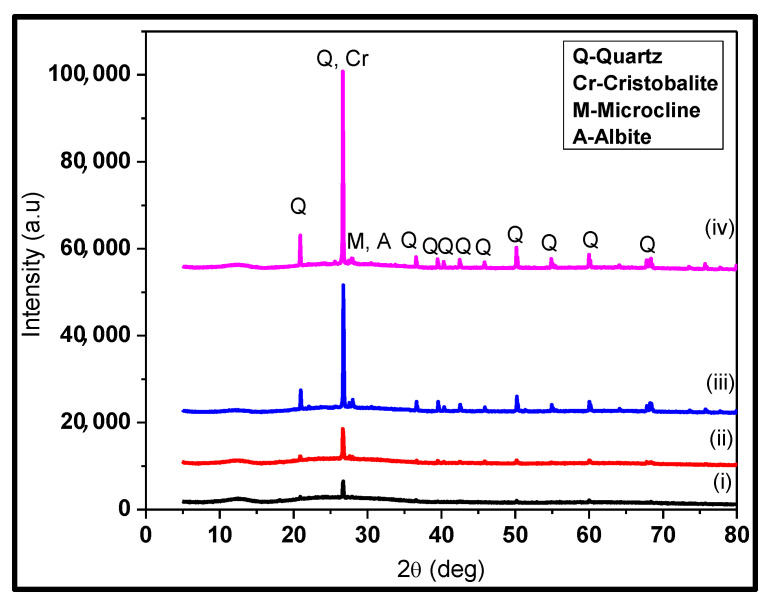
The XRD patterns for bagasse (i) SCBA CA leached (ii) CA-TPAH silica calcined bagasse (iii) SCBA L-Cys leached and (iv) L-cys-TPAH silica.

The XRD patterns presented in Figure 1 show the calcined bagasse (i) SCBA-CA leached Figure 1 (ii) CA-TPAH silica, Figure 1 (iii) SCBA L-Cys leached and Figure 1 (iv) L-cys-TPAH silica., revealed diffraction peaks at 2 theta angles of 20, 26, 36, 39, 50, and 59 corresponding to the crystalline phases of silica. The diffraction peak at 2theta angle 26 reveals that the extracted and calcined sugar cane bagasse consists of silica nanoparticles with a 101 cubic phase crystalline structure. Similar observations have been observed by Katare, V.D. and Madurwar, M.V. (2017), who revealed crystalline peaks of silica at 2θ = 26 [19]. This observation confirms the successful extraction of silica nanoparticles from sugar cane bagasse from our samples [1,19,20,21]. 

Figure 1 shows the XRD spectra of samples treated with Figure 1 (i), Figure 1 (ii) citric acid and Figure 1 (iii), Figure 1 (iv) L-cysteine hydrochloride monohydrate. It is quite evident that the characteristic peak at 2θ = 26 of quartz is associated with crystalline silica, and is present in both samples. However, the low intense peak for quartz crystalline peak on Figure 1 (i) reveals the weak leaching efficiency of citric acid as compared to Figure 1 (iii) L-cysteine hydrochloride [19,20,21,22].

It is worth noting that the intense peak at 2*θ* = 26 is characteristic of Quartz silica (JCP phase from ICDD: 01-083-0539) [20]. Furthermore, the crystalline silica is slightly more intense in (iv) than in (ii) [22]. The synthesized silica presented other peaks characteristic of cristobalite, which forms at higher temperatures indicating varied phases of silica in the sample [20]. Additionally, microcline (KAlSi_3_O_8_) and albite (NaAlSi_3_O_8_) minerals were also identified for the as-prepared silica shown in the diffraction patterns of low and weak peaks [21,22,23]. As mentioned earlier, the diffraction peaks at 2θ = 20, 26, 36, 39, 50, and 59 show that silica is highly crystalline mostly in a quartz form [24]. The average diameter sizes were determined to be approximately 26 nm and 29 nm using Scherrer’s formula for silica acid-leached with CA and L-Cys, respectively. The crystallite size was estimated by Scherrer’s formula as shown in Equation (5) below:

(5) $$Dp=\frac{K\lambda }{\beta \cos\theta }$$

whereby *Dp* is the Average crystallite size (nm), and *K* is known as the Scherrer constant. The *K* values vary from 0.68 to 2.08, and preferably *K* = 0.94 is utilized for spherical crystallites with cubic symmetry and λ-X-ray wavelength. For mini XRD, CuKα the *λ* = 1.54178 Å is normally desired. Additionally, *θ* is the peak position and β is identified by FWHM (full Width at Half Maximum) of XRD peak determined from one half of 2*θ*.

#### 3.1.2. FTIR Analysis

**Figure 2:**
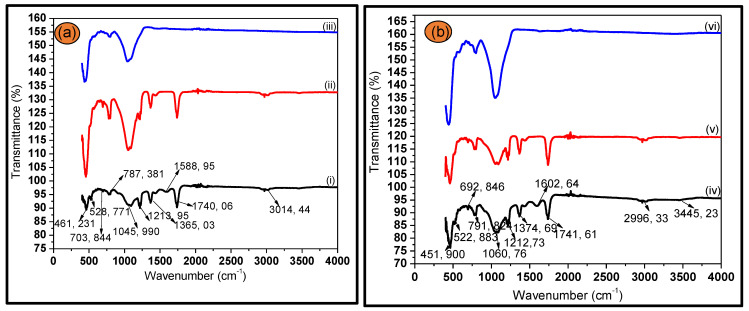
FTIR spectra of (**a**) SCBA (i) CA leached, (ii) calcined, (iii) silica and (**b**) SCBA (iv) acid-leached with L-Cysteine Hydrochloride, (v) calcined and (vi) Silica.

FTIR spectral analysis identified key functional groups existing in the bagasse ash, the leached samples and the nano-silica, which was operated at room temperature with wavelength range of 400–4000 cm^−1^. Figure 2, shows the FTIR analysis of the prepared Figure 2a (iii) silica, and exhibit bands at 461.231 cm^−1^, 787.381 cm^−1^ and 1045.99 cm^−1^ correspond to the Si~O~Si bending vibration, Si~O~Si stretch vibration and Si~O~Si stretching vibration, respectively. Interestingly, the evolution and disappearance of functional groups and the appearance of more prominent groups reveal the incorporation of new functionalities on our samples. The carbonyl group band is present in both (a) (i), (b) (iv) leached samples and (a) (ii), (b) (v) calcined samples however not observed for the silica spectra of both samples in (a) iii and (b) vi, which confirms the successful formation of silica groups, predominant in the material [1,24,25,26,27].

Similarly, the results obtained for as-produced (vi) silica in Figure 2b showed identical behavior of new functionalities. The bands at 451. 900 cm^−1^, 791.803 cm^−1^ and 1060.76 cm^−1^ are attributed to the Si~O~Si bending vibration, the Si~O~Si stretch vibration and the Si~O~Si stretching vibration [1,28]. 

Despite that, the silica in Figure 2b (vi), showed a highly narrow and pronounced band at around 1060.76 cm^−1^ which is characteristic of crystalline silica, confirming the results obtained from XRD diffractogram(s). Additionally, the band at 3014.44 cm^−1^ corresponded to C–H stretch in (a) i and ii, respectively, whereas in (b) iv and v, the C–H band is at 2996.33 cm^−1^. The weak absorption band for hydroxyl group (OH) exhibited weak is observed for treated bagasse with L-Cys in (b) iv, v at around 3445.23 cm^−1^ [26,27,28].

#### 3.1.3. SEM-EDX Analysis

The EDX results confirm that acid treatment is an efficient tool to minimize impurities from the increment (wt%) of Si matter in the matrix, and thus the extraction of silica from sugar cane was successful. In addition, the results indicate that TPAH is a useful organic solvent for extraction of silica from sugar cane bagasse ash acid treated with L-cysteine hydrochloride monohydrate. 

It can be observed that the morphology of powders from ash to silica and the selective removal of the synthesis residues using acid solution resulted in the formation of silica material with high composition, most importantly silica from ash-treated with L-cysteine hydrochloride monohydrate [29]. This may be due to the chloride present in the acid which was sufficient for the significant removal of metallic impurities as compared to the silica from ash treated with citric acid.

**Figure 3:**
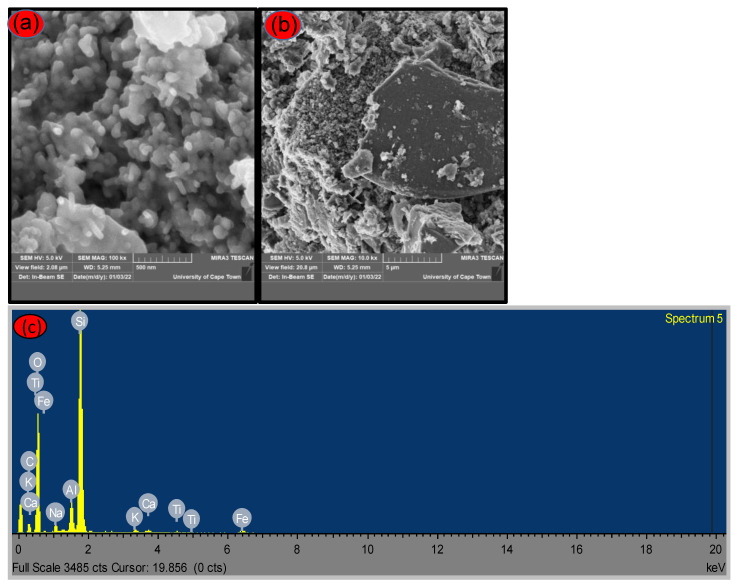
SEM images presentation of (**a**) leached silica (low magnification), (**b**) leached silica (high magnification) and (**c**) SEM-EDX of as-synthesized CA-TPAH silica treated with citric acid.

The surface morphologies of silica produced from SCBA were determined by SEM, and the SEM images are shown in Figure 3. The SEM images show that the samples in Figure 3a,b consist of agglomerates of irregular sizes, as well as the presence of some spherical particles distributed in the network of pores during silica synthesis, as there are irregular sizes of rice-like and spherical shape [27,28,29]. The morphological changes are in line with the surface characteristics analysis (FTIR), where the introduction of Tetrapropyl Ammonium Hydroxide on the calcined SCBA increased the formation of silica.

The average particle size from the SEM analysis was determined to be in the range of 200–500 nm, whereas the crystallite size estimated from XRD had an average diameter in the range 26–29 nm using the Scherrer formula.

SEM-EDX was conducted to study the composition of the synthesized silica using two different organic acids. Figure 3c, shows the EDX for the synthesized silica acid-treated with citric acid, the presence of metallic impurities beside the residual carbon and a more intense peak associated with silicon.

Table 1 below shows the elemental composition of bagasse ash, acid treated and silica. It is worth noting that the elemental Si composition in silica increases significantly, from raw-SCBA 1.78 wt% and 7.69 wt% for SCBA CA leached to 26.46 wt% and from 8.89 wt% to 34.7 wt% for SCBA L-cys leached.

The tabulated results above exhibit the morphology of powders from the ash to the extracted silica. It can be observed that the selective removal of the synthesis residues using acid solution resulted in the formation of a silicon with high composition, most importantly for silica from ash-treated with L-cysteine hydrochloride monohydrate [30]. This may be due to the chloride present in the acid which was sufficient for the significant removal of metallic impurity as compared to the silica from ash-treated with citric acid. Additionally, Aluminum (Al) and Iron Fe were found to be predominant at approximately 3.84 wt% and 2.25 wt%, respectively. 

**Figure 4:**
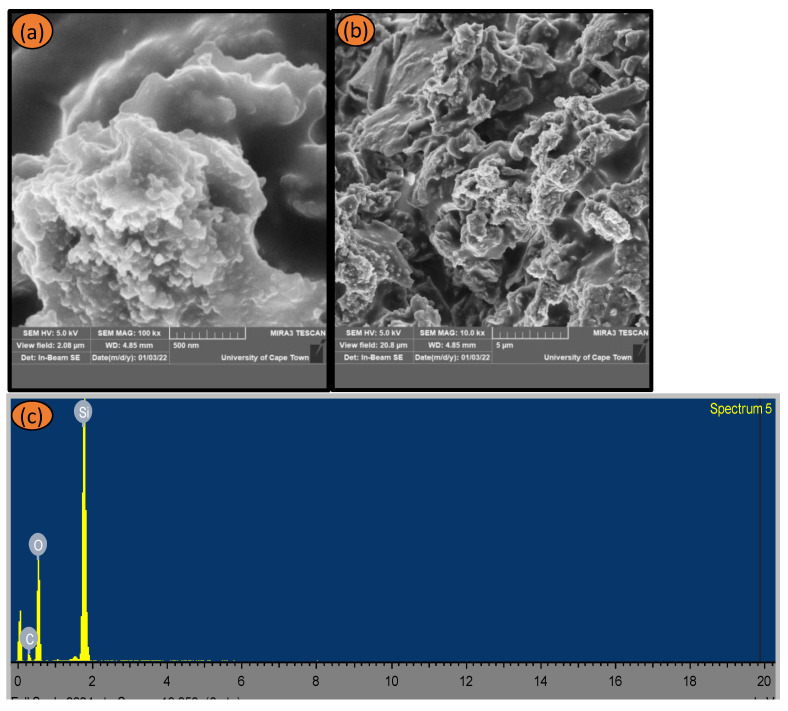
SEM micrograph presentation of (**a**) leached silica (low magnification), (**b**) leached silica (high magnification) and (**c**) SEM-EDX of as-synthesized L-cys-TPAH silica treated with L-cysteine Hydrochloride.

Figure 4a,b presents SEM micrographs of leached silica with L-cysteine hydrochloride monohydrate. The images revealed that silica nanoparticles are randomly distributed and clustered with a non-uniform and rough surface. The SEM-EDX of the as-prepared L-cys-TPAH silica is shown in Figure 4c, with the elemental composition predominantly silicon and oxygen with a very considerable amount of carbon present in the material.

The SEM-EDX exhibited fewer impurities in the treated sample(s), for L-cysteine hydrochloride monohydrate, which indicates the efficacy of acid-pretreatment(s) in reducing these metallic impurities as reported in [30]. 

#### 3.1.4. Thermal Analysis

**Figure 5:**
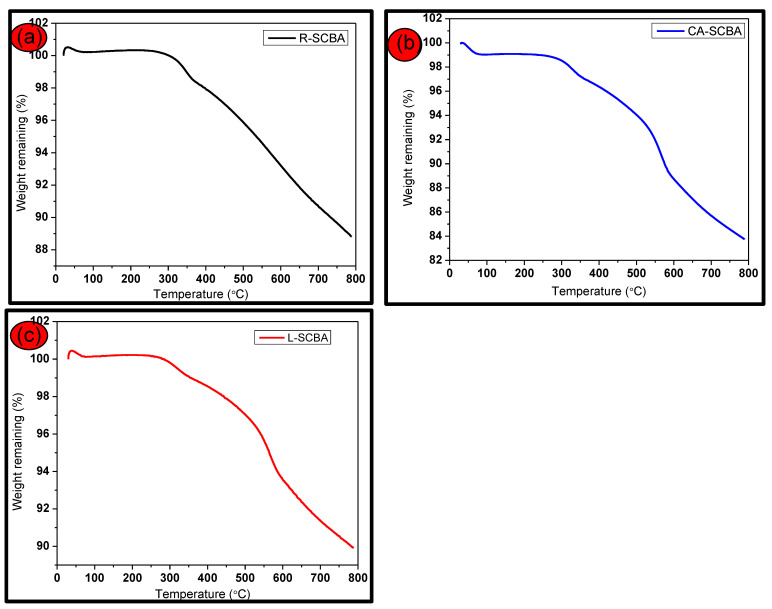
TGA distributions of (**a**) raw SCBA, (**b**) SCBA CA leached (**c**) SCBA L-cys leached respectively.

A thermogravimetric analysis (TGA) was conducted to investigate the thermal properties of the sugarcane bagasse ash from room temperature to 800 °C. This study essentially provided information on the pyrolysis/calcination temperatures and not the duration of the process. The thermogravimetric analysis in Figure 5 shows the thermal characteristics of (a) raw SCBA, (b) SCBA CA leached (c) SCBA L-cys leached respectively.

The thermal characteristics of the sugarcane bagasse make it quite evident that the volatilization stage took place where hemicellulose, cellulose and lignin decomposition occurred [31,32]. Their weight loss, shown in Figure 5, displays three main stages of dehydration, volatilization and carbonization, mostly for treated ash with CA and L-Cysteine, respectively. The TGA curves show that the evolution of thermal behavior started with the first stage of moisture drying occurring at 250 °C in all samples, owing to the crystallization in the interior part of the structures and light volatiles present in sugarcane bagasse ash [33]. 

The volatilization stage involves the decomposition of hemicellulose at approximately 180 °C and 340 °C, with the cellulosic breakdown occurring around 250 °C to 450 °C [34,35], whilst the lignin breakdown begins at 200 °C and goes to 800 °C [35]. Moreover, the rapid weight loss shown on the TGA curve (a) can be attributed to hemicellulose and cellulose decomposition. Interestingly, TGA curves for the pretreated samples (b) and (c) reveal rapid weight loss with three distinctive stages, which is similar to the results reported in [36,37]. 

The TG curves revealed a total mass loss of 12%, 18% and 11%, respectively, making the pretreatment with 5% citric acid solution the best organic acid for efficient leaching, as reported earlier in other studies. No further weight loss was observed, indicating the thermal stability of nanosilica present in the samples, which constitute about 84% in mass, as reported earlier in [38,39].

#### 3.1.5. Nitrogen Physisorption Analysis

The textural properties of scba nano-silica(s) were evaluated using the N_2_ adsorption-desorption method and the results are summarized in Table 2 below.

**Figure 6:**
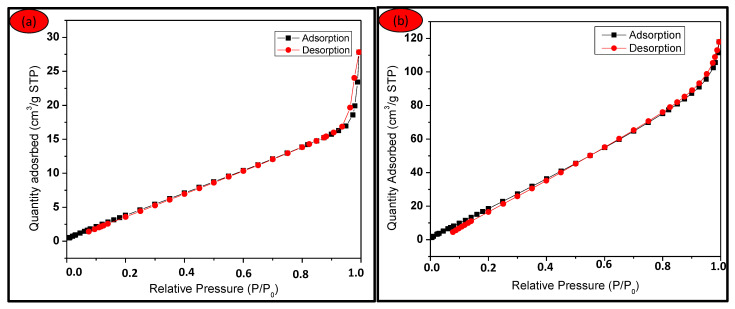
N_2_ adsorption-desorption isotherm for (**a**) SCBA nano CA-Silica and (**b**) SCBA nano L-cys Silica.

The BET_SSA_, pore volume and pore diameter for SCBA for nano CA-Silica were 21.6511 m^2^/g, 0.04312 cm^3^ and 8 nm, respectively, while BET_SSA_, pore volume and pore diameter for SCBA nano L-cys Silica is 116.005 m^2^/g, 0.1828 cm^3^/g and 6 nm, respectively. For CA-Silica displayed in Figure 6a, the amount of adsorbed gas increases steadily with increasing P/P_0_ ratio at the lower P/P_0_ regions, while it increases significantly for L-cys Silica with increasing P/P_0_, as shown in Figure 6b. This is usually attributed to monolayer and multilayer adsorption, as reported in [38,39].

For the SCBA nano silica isotherm in Figure 6a, the hysteresis loop is observed at a range of 0.9 < P/P_0_ < 1.0, essentially associated with capillary condensation taking place in the mesopores. Furthermore, the loop at the high P/P_0_ side has been reported to be related to large pores. The little loop at Figure 6b observed at range of 0.2 < P/P_0_ < 0.4 is also due to the capillary condensation inside the mesoporous structure, however no loop at the higher P/P_0_ region is observed for this synthesized silica (SCBA nano L-cys Silica) [38,39,40]. 

## 4. Conclusions

Physical characteristics and quantitative elemental composition of silica produced from sugarcane bagasse ash were investigated using FTIR and SEM-EDS. Thermal properties were studied using TGA, and the crystalline nanostructure was studied by XRD analysis. The average diameter sizes were determined to be approximately 26 nm and 29 nm for silica acid-leached with CA and L-Cys, respectively.

This study has shown that sugarcane bagasse ash contains highly crystalline silica which could be affected by leaching. The as-synthesized ca-silica and L-cys-silica of the extracted crystallite size had an average diameter of 26 nm and 29 nm, respectively, in addition to a specific surface area of 21.6511 m^2^/g and 116.005 m^2^/g for ca-silica and L-cys silica, respectively. The Infrared (IR) spectra showed peaks at 461.231 cm^−1^, 787.381 cm^−1^ and 1045.99 cm^−1^, which corresponds to the Si~O~Si bending vibration, the Si~O~Si stretch vibration, and the Si~O~Si stretching vibration, respectively. It was established that acid pre-treatment of the bagasse was more affected by concentration and time than temperature.

Based on the findings of this experimental study, the major component of SCBA is $SiO_{2}$ from the elemental composition with the O and Si elements displaying very high percentages. The ash is a predominantly carbonaceous material. The methodology also showed that with higher calcination temperatures other metallic impurities form, whereby Al and Fe were found in minor quantities after Si. After the synthesis of bio-silica, the structural, morphological and thermal properties were studied. The advantage of this finding is the green method of utilizing citric acid, L-cysteine hydrochloride monohydrate for acid treatment. IN addition, TetraPropylAmmonium Hydroxide monohydrate was used as an extraction solvent. Furthermore, the use of organic chemicals proved successful for the current study and are eco-friendly.

## Figures and Tables

**Table 1:** Chemical analysis of sugarcane bagasse composition acid treated with citric acid (ca) and L-cysteine hydrochloride monohydrate (L-cys).

Element	SCBA-Raw (wt%)	SCBA-Leached@CA (wt%)	Silica@TPAH-CA (wt%)	SCBA-Leached@L-cys (wt%)	Silica@TPAH-Lcys (wt%)
Carbon (C)	74.92	51.85	10.83	46.12	10.24
Oxygen (O)	22.45	28.4	53.39	42.29	55.05
Silicon (Si)	1.78	7.69	26.46	8.89	34.7
Potassium (K)	0.26	-	0.69	0.06	-
Calcium (Ca)	0.59	0.15	0.96	-	-
Sodium (Na)	-	-	1.09	-	-
Iron (Fe)	-	-	2.25	-	-
Titanium (Ti)	-	-	0.47	-	-
Aluminum (Al)	-	0.3	3.84	2.53	-
Fluorine (F)	-	11.61	-	-	-
Sulfur (S)	-	-	-	0.1	

**Table 2:** Textural properties of SCBA nano CA-Silica and L-cys Silica.

Sample ID	BET_SSA_ (m^2^/g)	Vp (cm^3^/g)	Dp (nm)
CA-Silica	21.6511	0.04312	8
L-cys Silica	116.005	0.1828	6

## Data Availability

Data used for this study is reported and can be found within the article.

## References

[1] Mohd N.K., Wee N.N.A.N., Azmi A.A. (2017). Green synthesis of silica nanoparticles using sugarcane bagasse. AIP Conference Proceedings.

[2] Herzog H., Golomb D. (2004). Carbon capture and storage from fossil fuel use. Encycl. Energy.

[3] Salem S.S., Fouda A. (2021). Green synthesis of metallic nanoparticles and their prospective biotechnological applications: An overview. Biol. Trace Elem. Res..

[4] Rodríguez-Félix F., López-Cota A.G., Moreno-Vásquez M.J., Graciano-Verdugo A.Z., Quintero-Reyes I.E., Del-Toro-Sánchez C.L., Tapia-Hernández J.A. (2021). Sustainable-green synthesis of silver nanoparticles using safflower (*Carthamus tinctorius* L.) waste extract and its antibacterial activity. Heliyon.

[5] Del-Toro-Sánchez C.L., Rodríguez-Félix F., Cinco-Moroyoqui F.J., Juárez J., Ruiz-Cruz S., Wong-Corral F.J., Borboa-Flores J., Castro-Enríquez D.D., Barreras-Urbina C.G., Tapia-Hernández J.A. (2021). Recovery of phytochemical from three safflower (*Carthamus tinctorius* L.) by-products: Antioxidant properties, protective effect of human erythrocytes and profile by UPLC-DAD-MS. J. Food Processing Preserv..

[6] Rodríguez-Félix F., Corte-Tarazón J.A., Rochín-Wong S., Fernández-Quiroz J.D., Garzón-García A.M., Santos-Sauceda I., Plascencia-Martínez D.F., Chan-Chan L.H., Vázquez-López C., Barreras-Urbina C.G. (2022). Physicochemical, structural, mechanical and antioxidant properties of zein films incorporated with no-ultrafiltred and ultrafiltered betalains extract from the beetroot (*Beta vulgaris*) bagasse with potential application as active food packaging. J. Food Eng..

[7] Rodríguez-Félix F., Graciano-Verdugo A.Z., Moreno-Vásquez M.J., Lagarda-Díaz I., Barreras-Urbina C.G., Armenta-Villegas L., Olguín-Moreno A., Tapia-Hernández J.A. (2022). Trends in Sustainable Green Synthesis of Silver Nanoparticles Using Agri-Food Waste Extracts and Their Applications in Health. J. Nanomater..

[8] Farirai F., Mupa M., Daramola M.O. (2021). An improved method for the production of high purity silica from sugarcane bagasse ash obtained from a bioethanol plant boiler. Part. Sci. Technol..

[9] Chung I.M., Park I., Seung-Hyun K., Thiruvengadam M., Rajakumar G. (2016). Plant-mediated synthesis of silver nanoparticles: Their characteristic properties and therapeutic applications. Nanoscale Res. Lett..

[10] Dos Santos R.M., Neto W.P.F., Silvério H.A., Martins D.F., Dantas N.O., Pasquini D. (2013). Cellulose nanocrystals from pineapple leaf, a new approach for the reuse of this agro-waste. Ind. Crops Prod..

[11] Lauwers A.M., Heinen W. (1974). Bio-degradation and utilization of silica and quartz. Arch. Microbiol..

[12] Anuar M.F., Fen Y.W., Zaid M.H.M., Matori K.A., Khaidir R.E.M. (2020). The physical and optical studies of crystalline silica derived from the green synthesis of coconut husk ash. Appl. Sci..

[13] Chruściel J.J., Leśniak E. (2015). Modification of epoxy resins with functional silanes, polysiloxanes, silsesquioxanes, silica and silicates. Prog. Polym. Sci..

[14] Ndububa E.E., Nurudeen Y. (2015). Effect of guinea corn husk ash as partial replacement for cement in concrete. IOSR J. Mech. Civ. Eng. (IOSR-JMCE).

[15] Yi D.K., Lee S.S., Papaefthymiou G.C., Ying J.Y. (2006). Nanoparticle architectures templated by SiO_2_/Fe_2_O_3_ nanocomposites. Chem. Mater..

[16] Norsuraya S., Fazlena H., Norhasyimi R. (2016). Sugarcane bagasse as a renewable source of silica to synthesize Santa Barbara Amorphous-15 (SBA-15). Procedia Eng..

[17] Aprianti E., Shafigh P., Bahri S., Farahani J.N. (2015). Supplementary cementitious materials origin from agricultural wastes–A review. Constr. Build. Mater..

[18] Katare V.D., Madurwar M.V. (2017). Experimental characterization of sugarcane biomass ash–A review. Constr. Build. Mater..

[19] Thomas B.S., Yang J., Mo K.H., Abdalla J.A., Hawileh R.A., Ariyachandra E. (2021). Biomass ashes from agricultural wastes as supplementary cementitious materials or aggregate replacement in cement/geopolymer concrete: A comprehensive review. J. Build. Eng..

[20] Moayedi H., Aghel B., Nguyen H., Rashid A.S.A. (2019). Applications of rice husk ash as green and sustainable biomass. J. Clean. Prod..

[21] Khan N.A., Ibrahim S., Subramaniam P. (2004). Elimination of heavy metals from wastewater using agricultural wastes as adsorbents. Malays. J. Sci..

[22] Falk G., Shinhe G.P., Teixeira L.B., Moraes E.G., de Oliveira A.N. (2019). Synthesis of silica nanoparticles from sugarcane bagasse ash and nano-silicon via magnesiothermic reactions. Ceram. Int..

[23] Rahmat N., Sabali M.A., Sandu A.V., Sahiron N., Sandu I.G. (2016). Study of calcination temperature and concentration of NaOH effect on crystallinity of silica from sugarcane bagasse ash (SCBA). Rev. Chim..

[24] Shinohara Y., Kohyama N. (2004). Quantitative analysis of tridymite and cristobalite crystallized in rice husk ash by heating. Ind. Health.

[25] De Lima V.M.E., Barros L.C., de Melo A.A. (2021). Characterization of sugarcane bagasse ash (SBA) and its evaluation for use in alkali-activated slag mixtures. Cerâmica.

[26] Maza-Ignacio O.T., Jiménez-Quero V.G., Guerrero-Paz J., Montes-García P. (2020). Recycling untreated sugarcane bagasse ash and industrial wastes for the preparation of resistant, lightweight and ecological fired bricks. Constr. Build. Mater..

[27] Jagadesh P., Ramachandramurthy A., Murugesan R., Sarayu K. (2015). Micro-Analytical studies on sugar cane bagasse ash. Sadhana.

[28] El-Sayed S.A., Mostafa M.E. (2015). Kinetic parameters determination of biomass pyrolysis fuels using TGA and DTA techniques. Waste Biomass Valorization.

[29] He C., Giannis A., Wang J.Y. (2013). Conversion of sewage sludge to clean solid fuel using hydrothermal carbonization: Hydrochar fuel characteristics and combustion behavior. Appl. Energy.

[30] Trninić M., Jovović A., Stojiljković D. (2016). A steady state model of agricultural waste pyrolysis: A mini review. Waste Manag. Res..

[31] Çepelioğullar Ö., Pütün A.E. (2013). Thermal and kinetic behaviors of biomass and plastic wastes in co-pyrolysis. Energy Convers. Manag..

[32] Lv G.J., Wu S.B., Lou R. (2010). Kinetic study for the thermal decomposition of hemicellulose isolated from corn stalk. BioResources.

[33] Abdullah N., Sulaiman F. (2013). A comparison study on oven and solar dried empty fruit bunches. Technology.

[34] Aboyade A.O., Hugo T.J., Carrier M., Meyer E.L., Stahl R., Knoetze J.H., Görgens J.F. (2011). Non-isothermal kinetic analysis of the devolatilization of corn cobs and sugar cane bagasse in an inert atmosphere. Thermochim. Acta.

[35] Rafiee E., Shahebrahimi S. (2012). Nano silica with high surface area from rice husk as a support for 12-tungstophosphoric acid: An efficient nano catalyst in some organic reactions. Chin. J. Catal..

[36] Rafiee E., Shahebrahimi S., Feyzi M., Shaterzadeh M. (2012). Optimization of synthesis and characterization of nanosilica produced from rice husk (a common waste material). Int. Nano Lett..

[37] Yadav A.L., Sairam V., Srinivasan K., Muruganandam L. (2020). Synthesis and characterization of geopolymer from metakaolin and sugarcane bagasse ash. Constr. Build. Mater..

[38] Mantoura S. (2007). Reduce and replicate. Nat. Nanotechnol..

[39] Shen L., Guo X., Fang X., Wang Z., Chen L. (2012). Magnesiothermically reduced diatomaceous earth as a porous silicon anode material for lithium ion batteries. J. Power Sources.

[40] Li M., Dai Y., Ma W., Yang B., Chu Q. (2017). Review of new technology for preparing crystalline Silicon solar cell materials by metallurgical method. IOP Conference Series: Earth and Environmental Science.

